# Healthcare avoidance: a qualitative study of dental care avoidance in Germany in terms of emergent behaviours and characteristics

**DOI:** 10.1186/s12903-021-01933-1

**Published:** 2021-11-08

**Authors:** Isabell Gragoll, Lukas Schumann, Monique Neubauer, Christina Westphal, Hermann Lang

**Affiliations:** 1 Department of Operative Dentistry and Periodontology, Rostock University Medical Center, Strempelstraße 13, 18057 Rostock, Germany; 2 Institute for General Pedagogy and Social Pedagogy, University of Rostock, August-Bebel-Straße 28, 18055 Rostock, Germany; 3 Max Planck Institute for Demographic Research, Konrad-Zuse-Straße 1, 18057 Rostock, Germany; 4 Department of Extracorporeal Therapy Systems, Fraunhofer Institute for Cell Therapy and Immunology, Schillingallee 68, 18055 Rostock, Germany

**Keywords:** Healthcare avoidance, Dental care avoidance, Avoidance of the dentist, Avoidant behavior, Qualitative study, Typology

## Abstract

**Background:**

The treatment of acute pain is part of everyday dental practice. Often, these symptoms result from years of patients' inadequate or missing dental routines and lead to a reduction in the quality of life or health of the patients and to high costs for the health care system. Despite the enormous advantages of modern dentistry, many patients avoid going to the dentist. Therefore, the study aimed to determine the reasons and behaviours that cause patients to avoid visits to the dentist.

**Methods:**

We conducted semi-structured interviews with patients who had an above-average DMFT index and had been going to the dentist only irregularly for years. The sample participants were recruited from the northern German region of Mecklenburg-Western Pomerania. 20 individual interviews were recorded, transcribed verbatim and coded. We used a qualitative framework approach to code the transcripts in order to establish a consensus among the researchers. Ultimately, through discussions and reviews of the attributes and meaning of the topics, a typology could be established.

**Results:**

A typology of patients who avoid the dentist was developed. Four independent characteristic patterns of dentist avoidance could be developed: avoiding the dentist due to "distance" (type A; includes subtype A1 "avoiding the dentist due to negligence" and subtype A2 "dental avoidance due to neutralization"), "disappointment" (type B), "shame" (type C), and "fear" (type D). Using the typology as a generalised tool to determine the minimum and maximum contrasts, it was possible to capture the diversity and multidimensionality of the reasons and behaviours for avoidance. All patients had negative dental experiences, which had led to different avoidance patterns and strategies.

**Conclusions:**

The identified avoidance characteristics represent a spectrum of patients from Northern Germany who avoid going to the dentist. This is the first comprehensive study in Germany representing avoidance behaviour of dentist patients in the form of a typology. The results suggest that dentistry also needs qualitative research to better understand patient characteristics and provide direct access to patients who avoid regular dental visits. Thus, the results make a potentially fundamental contribution to the improvement of dental care and enrich its understanding.

## Background

Despite the increasing medical awareness in today's society and the benefits of modern medicine, numerous people try to avoid health care. At the same time, however, they are aware of the need for preventive measures [1]. This paradox is a well described problem at the national and international level, and has been addressed in the medical literature for various health care settings [2–5]. Dentistry in Germany is well aware of this problem, as about 7–10% of the population avoid visiting the dentist [6]. The abandonment of medical care inevitably affects the quality of life and health status of affected individuals [7, 8]. It has also serious consequences for the health care system in terms of expenditures [9], as the restoration of dental damage usually requires lengthy therapy and rehabilitation [10].

Despite the awareness of the problem of avoidance and its consequences, there have been only a few studies in medicine, particularly in dentistry, that explore the basic principles of patients' avoidance behaviour. Most of these studies are quantitative and limited to specific diseases and disciplines in human medicine [11–13]. There are also studies with large samples that explore overall health care utilization and influencing variables. Studies such as the HINTS study [14], the EPESE study [15], and the DMS V study [16] serve as essential sources of information on health data and health behaviour. Studies of this kind have been used for the assessment and specification of avoidance attitudes and access to the health care system in other areas [2, 4, 17]. Additionally, several behavioural models were created. The Health Belief Model [18] was one of the first models from a social science perspective to describe the influence of perceptions and beliefs on health behaviour. The Behavioural Model of Health Services Use [19] also describes the complexity of health behaviours.

In dentistry, various influencing variables and factors are associated with avoiding seeing a dental physician. It is known, for example, that certain social-demographic constellations influence people’s attitudes towards regular dental check-ups [20, 21]. In addition, some behavioural patterns of avoidance patients rely upon have already been described. For example, dental anxiety has been discussed many times [22–24], therefore it is now considered necessary to understand the development of anxiety throughout the lifespan [25]. Another aspect is embarrassment [26]. The phenomenon of dental indifference [27], which discourages patients from regular dental care as well as motivational behaviours [28] to encourage dental care routines have also been discussed.

Despite a wide variety of study approaches and models, only the isolated perspectives and experiences of individual patients regarding their avoidance behaviour have been successfully remedied. There is a lack of understanding of what influences specifically affect patients and why these ultimately lead to avoidance behaviours. As a consequence, many researchers point to the need for qualitative research to understand the complexities of avoidance [2, 29, 30]. The mere listing of significant numerical values does not lead to a better understanding, since the causes of the avoidance behaviour can only be accessed through the personal experiences of the affected patients. In addition, research into social interactions and health-related behaviours needs to be expanded to make possible interventions more feasible [1].

The present study starts at this point and uses a qualitative study design to investigate the reasons for avoiding visits to the dentist. Based on qualitative, guided interviews, the patients' statements and views are directly included in the analyses and a typology is developed. With the help of this typology, avoiding patients can be better classified and understood.

## Methods

### Participants and sampling criteria

The study was carried out under the leadership of the Dental Clinic of the University Medical Center Rostock. The aim was to enroll 30 patients for a qualitative, guided interview. In order to meet the target number of patients, patients from selected dental practices in the surrounding (listed in the declarations) area were also included. The study was approved by the Ethics Committee of the University of Rostock (registration number: A 2017- 0165). The sample was limited to the northern German region of Mecklenburg-Western Pomerania. The study is based on a consecutive sampling design. Between June 2018 and August 2019, we identified patients who received treatment or visited the dental pain service at one of the above facilities after an extended absence, thus meeting the inclusion criteria described below.

In general, we were looking for patients with a higher than average DMFT index, compared to the fifth German Oral Health Study [16] who had avoided a regular dental routine for years [31]. Patients with mental health problems were not included in the survey. Verification of inclusion criteria was ensured by reviewing the medical records of each participant.

Sampling criteria for inclusion in the qualitative patient survey study.

- Age: 18–70
- No manifest mental disorders
- DMFT index > 11.2 (adults, included 18-to 64-year-old participants) or > 17.7 (seniors included 65-to 70-year-old participants) based on 28 teeth (DMFT: the sum of decayed, filled and missing teeth) and oriented to the fifth German oral health survey DMS V with adjustment of the age limit
- The patient has not visited a dentist within the last two years or more and has not been treated for any of the symptoms associated with the present disease within the previous two years or more, or
- the patient did not adhere to the dentist's treatment recommendations during this period despite visits to the dentist, or
- the patient was under acute dental treatment only
- Dental phobia patients were excluded from the study
- Informed consent by the patient

Potential participants were contacted directly and informed about the study. If interested, the candidate was contacted by the interviewer and invited to a separate interview appointment. IG conducted the interview as a doctoral student in dentistry. Methodologically, the study was implemented under the comprehensive supervision of a social scientist specializing in qualitative studies. In addition, the study was guided throughout by a team of (dental) physicians and social scientists.

### Qualitative interviews

Before the actual interview began, the interviewees were given more background information about the project to set the scene. This included the opportunity for patients to ask questions. The interviewer explained to them that the focus of the study was to understand what had led to years of dental treatment avoidance, and emphasized the patient perspective was essential for this. The interviewer informed about the anonymity of the patient data and handed out a patient information sheet. All participants signed a consent form which also provided for the scientific publication of the research results in compliance with data protection regulations.

For the study, we relied on the guided interview method [32]. This method ensures that the interviewees can talk freely about their experiences while the interviewer can still steer the conversation. This provided the respondents with a flexible and accessible response space, which was necessary for the subsequent qualitative content analysis. The development of the guiding questions was based on a comprehensive literature review of the current state of research on health care avoidance with a dental focus. On this basis, we ensured that existing knowledge about physician avoidance behaviour was taken into account when creating the questions and that the results could be verified and qualitatively expanded accordingly [2, 4, 17, 20, 23, 26, 28, 30, 33]. Before the guide was finally used, it was pilot—tested.

The interviewer did not determine the length of the interviews’ in advance but let him-/herself be guided by the participants' verbal and non-verbal cues. In general, the interviews lasted between 30 and 60 min. All interviews were electronically recorded with patient consent and interviews were not repeated. After the interview, the interviewer filled out log sheets to record reflections and considerations, salient non-verbal aspects of the interview, the duration of the interview, the atmosphere, information about success or failure, possible disruptions and interruptions, and information gathered before or after the interview.

We chose a quiet environment for the interviews. Ideally, this was a room in the dental clinic. In rare individual cases, the interview was held in a quiet atmosphere at the subject's home. We tried to avoid having additional people present during the interview. After completing the interviews, the interviewer transcribed the digital recordings verbatim according to Kuckartz's transcription rules [34]. All personal data were encrypted.

### Qualitative analysis

Data collection and analysis were interwoven using grounded theory methodology according to the principle of theoretical sampling until the field was theoretically saturated [35]. For the evaluation of the transcribed individual interviews, the thematic qualitative text analysis procedure and the typifying qualitative analysis procedure according to Kuckartz were applied [34]. As part of the thematic qualitative text analysis, all of the collected transcript material was coded. This served the basic structuring of the data material and was a crucial preliminary work for the subsequent typification process.

The MAXQDA software (MAXQDA 2018, https:www.maxqda.com) was used as the coding programme for the analyses. The type formation was based on categorisation. Categorisation served to condense the data and accurately classify the transcript content. Deductive categories were already derived in advance from the created guideline. From the text material itself, inductive categories were developed. The deductive and inductive categorisation led to the formation of main categories and sub-categories. This resulted in a hierarchically structured system of categories. To allow transparent interpretation of the categories and comparability among the researchers, they were clearly defined in memos [34] To provide the reliability of the assigned categories, study team members (IG, MN, CW, UZ) continually compared selected code associations until a consensus was reached [36]. Using the category system developed, we were able to identify relevant comparative characteristics among the participants.

### Type formation

The idea of our peer debriefing research process of the typology was to take place in a communicative and open environment in which the subjects' experiences could be examined and studied as value-free as possible [37]. To ensure the quality of the research, peer debriefings and reflections on the research field were carried out in advance to avoid possible prejudices. This way, stereotyping, which is understood as a rigid impression due to hasty judgment instead of analysis and review, could be avoided [38]. We aimed to create a value-free typology of different dentist avoidance patterns based on the patients' statements. In the end, this results in a jointly and openly developed problem analysis.

Type formation has a long tradition in the social sciences, in particular dating back to Max Weber and Alfred Schütz [37]. Udo Kuckartz was one of those scientists who, building on this, reformulated the controllability of type formation. We based our typification on Kuckartz's qualitative content analysis in order to use a well-described method for typfication and to be able to construct types in a methodically controllable way [34]. In the first phase of type construction, we determined relevant dimensions for the intended typology [34]. Since we had a multidimensional feature space, we relied on polythetic typing according to Udo Kuckartz [34]. After agreeing on the categories in the research team, we developed short portraits of each individual case based on the codings. The case construction was inductive. Only the broad categories were already determined by the guideline.

Consequently, the cases were grouped in such a way that they were clearly distinguishable from other patterns and groups. Polythetic typing also resulted in trait heterogeneous combinations. This means that some individuals belonging to one type are not identical with respect to the features of the feature space, but are still similar [34]. Thus in our scheme, a type consisted of several individual cases which are very similar to each other.

In two rounds of collegial meetings, our team discussed the resulting typology framework. Subsequently, the cases created the typology. The aim of the typology was to depict types with maximum heterogeneity and the greatest possible internal homogeneity [37]. As specified by Kuckartz, the individual cases were unambiguously assigned to the appropriate types [33]. Finally, we concluded the typology formation with the precise classification of each case by consensus of all researchers involved in the project.

### Qualitative strengths

Our study was guided by expertise from an interdisciplinary team of researchers covering the following disciplines: Dentistry, Dental Care and Oral Health (IG, LS, HL), Sociology and Education (UZ), Clinical and Demographic Health Research (CW), and Qualitative Methodology (MN).

Throughout the study period, regular collegial exchanges took place with the researchers involved in the study. The methodological evaluation approaches according to Kuckartz, were strictly followed to ensure rule-governedness. Post-interview protocol sheets and case summaries increased the transparency of the collected data material. Finally, direct quotes from the individual interviews supported the interpretations.

For data collection, we adhered to the principle of theoretical saturation within the framework of Grounded Theory methodology [35]. In this respect, data collection was terminated when no more new topics could be identified through the interviews and the field to be researched had thus been exploited. That is, the subject area under investigation had developed to the point where no further information could be obtained through additional analysis.

## Results

A total of, 60 potential study participants were identified and contacted, of which 34 showed up for the interview. The number of contacted subjects who did not show up for the interview consisted of individuals who either did not respond to being contacted or had agreed to participate but then canceled multiple appointments (Table 1). This illustrates the sensitivity of the topic and the associated difficulties in accessing data.

**Table 1:** Recruitment frequency

Number of contacted study participants	n = 60
Contacted persons, without response to the request	n = 13
Did not show up for the interview after verbal commitment and an appointment was made	n = 13
Recruited participants	n = 34
Thereof male participants	n = 21
Thereof female participants	n = 13

Of the total of 34 interviews conducted, we included 20 in the overall analysis (Table 2). The reasons for exclusion from the qualitative analysis were missing answers to the questions or isolated deviations from the defined sampling criteria that only became apparent during the interview.

**Table 2:** Characteristics of the participants included in the final analysis

Number of participants included in the final analysis	n = 20
Thereof male participants	n = 11
Thereof female participants	n = 9
Average age of the participants	43.3
Average DMFT index adults 18- to 64- year- old	19.7
Average DMFT index seniors 65- to 70- year- old	23.3

The values of the DMS V (2016) were used as a basis. In younger adults (35-to 44-year-olds), the caries index was 11.2, and in younger seniors (65-to 74-year-olds), 17.7. In our study, the range of younger adults was expanded to include all participants from 18 years up to and including 64 years of age in order not to omit any participants. For the younger seniors (65-to 70-year-olds), subjects were only included up to the age of 70, as our sampling criteria only reached up to this age

By constantly comparing and contrasting the individual cases, we were able to uncover a typology of four distinct patterns that reflect the range of dentist avoidance (Table 3). These are dentist avoidance due to:

1. distance (type A). Type A includes subtype A1 "avoiding the dentist through negligence" and subtype A2 "dental avoidance through neutralization"),
2. disappointment (type B)
3. shame (type C)
4. fear (type D)

**Table 3:** Typology of dentist avoidance characteristics

	How is the avoidance attitude identified?	What are the main reasons for avoidance?	Avoidance mechanism
Avoiding the dentist due to distance (A)	Targeted displacement and distancing from dental care	Personal negligence	Avoidance and displacement
Avoiding the dentist due to negligence (subtype A1)	Repression of avoidance problems	Own lack of motivation and suppression of existing problems in certain stages of life or by treatment processes of a particular stage of life	Negligence leads directly to insufficient oral hygiene, which in turn leads to more disruptive dental treatments; this leads to the reinforcement of avoidance and negligence
	Repression of confrontation with avoidance attitudes and consequences		
Dental avoidance due to neutralization (subtype A2)	Use of statements to explain the absence and neutralisation of possible reasons for avoidance	Own discussion of multicausal factors	Listing of avoidance causes to explain abstinence and distancing oneself from responsibility
Avoiding the dentist due to disappointment (B)	Questioning the dentist and his intentions	Perceived lack of dental advice and care	The difficulty to trust fuels the avoidance mindset
	The feeling of being a means to an end	Distrust, helplessness, disappointment	
		feeling that the monetary approach takes precedence over the humanitarian approach	
Avoiding the dentist due to shame (C)	A deep sense of shame	Own shame and concern about condemnation	Avoiding confrontation with one's shame by avoidance
Avoiding the dentist due to fear (D)	Anxiety attitude up to mental and physical complaints	Fear of the dentist and dental treatments	Fear outweighs the importance of going to the dentist
		Fear of not respecting the fear attitude	

Retrospectively, all participants were able to name negative experiences with the dentist or dental treatments. These negative experiences triggered different behavioural patterns and ways of dealing with the existing problem in the respondents. Across all cases, it became evident that all types identified in the study had regularly suppressed dental visits for years. However, the avoidance and suppression strategies varied significantly, ranging from personal carelessness to avoiding dental visits out of fear.

### Type A: Avoiding the dentist due to distance

Distanced patients suppress a visit to the dentist for years and have developed a distinct lack of concern about visiting the dentist. The distant type can be further divided into two subtypes, depending on how the attitude is handled: through negligence (subtype A1) and avoiding the dentist through neutralization (subtype A2). Whereas the subtype A1 avoids the dentist primarily because of an adolescent process, subtype A2 avoids the dentist because of a process which occurs more likely later in life. Patients who avoid the dentist through neutralization try to conceal their avoidance with various arguments. In contrast, negligence patients tend to report freely about their carelessness and regret it, too, retrospectively.

### Subtype A1: Avoiding the dentist due to negligence (n = 3)^1^

Lack of understanding of the importance of dental treatment and a lack of self-motivation are characteristic of this type.

This type associates the negligent attitude with a particular stage of life (A1.1) or a negative experience with a dentist at a particular stage of life.

The current lack of interest in oral hygiene leads to ignoring existing problems. As a result, dental health deteriorates due to poor dental hygiene. The visit to the dentist, which is indispensable due to the need for treatment, leaves an increasingly negative impression on the patient, and thus, reinforces the attitude of avoidance.

Well, youthful recklessness, I'd say. I used to like to put things off, really: "Oh, I'll go next week." And then a week became a month, and then it was completely gone." […] "Hm, well, maturity was lacking a bit, I'd say. One was still so careless. Youthful recklessness, I'd say. That was the main reason." […] "And then a lot of things had to be done because some teeth were broken and that wasn’t such a nice experience. And so I went even less, due to the bad experience with the dentist and then, of course, it didn't get any better with my teeth (male participant, age 20).

At some point, fixed braces were added, and that's when the problems really started. Because of those brackets that you have on your teeth. As a teenager, you are sloppy. You don't keep up with it, and when the braces come off, the teeth looked unattractive." […] "[…], so somehow it was my own sloppiness. I just didn’t take care of myself (female participant, age 27).

The possible consequences of avoiding the dentist are not considered. Going to the dentist is made more difficult by the self-awareness of negligence and the fear of possible confrontation. In retrospect, the patients communicate this openly and admit their negligence self-critically.

I never before thought about the fact that you may somehow have effects from it lateron" […] "If I’d just thought a little bit, yes, thinking ahead. Such effects weren’t really necessary. It's just common sense that it should have been done (female participant, age 27).

### Subtype A2: Dental avoidance through neutralization (n = 4)^1^

Due to negative experiences with a dentist, this type feels the need to postpone dental visits to avoid another confrontation.

To this end, arguments are sought to explain the absence from a dental appointment As an excuse, the patients mentioned various reasons why a visit to the dentist had been not possible. For example, working hours are cited as a limiting time factor that does not allow the patient to keep a dental appointment.

[...] but at the moment it's just not possible for me to go to the clinic here [...] because of the seasonal business (female participant, age 25).

Another example is the statement that it is simply too difficult to find a new dentist.

No, and as my dentist didn’t reopen her practice afterwards, we would have had to look for someone else (female participant, age 62).

Also contradictory statements are found during the interview that later turn out to be part of a justification strategy and are used to neutralize the process. The reason originally given for the avoidance becomes an excuse or shows a consequence of one's own wrongdoing.

I mean, if I’d wanted to, I could’ve done it, right? Somehow, or maybe even in the evening. [...] That’s when I only worked on the late shift until ten in the morning, but then I’m sure that complacency and laziness were also involved [...] (female participant, age 62).

There is also an attempt to reinforce or invalidate one's own misconduct by giving other reasons for avoidance.

Ugh, the time factor was also quite decisive. Just through training, then the season, whatever, and when you really have time it’s like 'Do you feel like it? Are you going to do that now, or wouldn’t you rather do something else?" […] "With me, it's really just this time factor. And if not time, then the pleasure factor. 'Do you want to get on the bus now? Do you want to go and sit there for two hours?' Okay, I know that it should have been a must, I knew that at the time. but then there's this laziness. That you say: "No, I'm not going, I'm going to lie on the settee. I'm not doing anything today," and that there was no insistence. I’m not blaming my parents, but they should have said: "You go now! I'm going with you!" or whatever. You see, insistence was simply missing [...] (female participant, age 25).

Patients use avoidance reasons to distance themselves from the responsibility for their dental condition. At the same time, they distance themselves from possible reproaches and their own guilt about the current situation.

### Type B: Avoiding the dentist due to disappointment (n = 7)^1^

Disappointed patients feel that the dentist has ignored their ideas and assumptions. Perceived deficits in the provision of information about dental treatments have led to an apparent distrust in dentists and dental treatments. As a result, these are increasingly critically questioned and doubted.

[…] many people just convey the feeling that they just want your money. So, that is what influences many, it also influences me, of course. [...] then the dentist wants to fiddle around with your teeth or wants to pull teeth or he insists on crowns or something else, although there’s no need for it. At least sometimes, you yourself can’t judge whether it’s true or not (male participant, age 33).

Also, the disappointed patients feel that their needs are ignored and respected insufficiently. They often think they had been presented with a fait accompli and had not been included in the treatment process.

That was always the case [...] this: "Must be done immediately. The why always remained so vague in space and that was it then, too, for me, I just stopped going there (male participant, age 33).

Respondents state that they feel subordinated to the dentist's decision. Perplexity, disappointment, and an enormous loss of trust are the results. From the patient's point of view, s/he serves only as a means to an end and is subject to the dentist's financial considerations.

I'm the loser here who comes in with a health insurance card where there's not a lot of money to be made, or so I guess. Anyway, the interest is simply not there. [...] I don't want people to dealing with me go over my head, but rather that they take my concerns and wishes serious. [...] that one also looks whether there is somehow an alternative, perhaps a compromise. Or explains what can be done, or asks how can I help you? Nothing of the sort there, not on your money (male participant, age 59).

### Type C: Avoiding the dentist due to shame (n = 2)^1^

A profound feeling of shame characterises this type. The main reason for this is the patient’s fear that the dentist might judge him/her negatively. The feeling of guilt can have various origins. For example, respondents feel ashamed of their behaviour in the dentist’s chair.. However, the shame is base, primarily on the current condition of the dentition and concerns with the associated public image.

And afterwards, when my teeth were broken, I didn't dare go, because I was ashamed to show up at the dentist’s looking like that (female participant, age 62).

The feeling of shame increases due to the avoidance and the resulting progression of aesthetic defects. The mental confrontation with shame is also present outside of dental treatments and, therefore, also represents a psychological burden to the interviewees. The aesthetic defects cause mood fluctuations, which can increasingly limit the patient’s quality of life.

Quite a lot. So actually, I would say 90% of the time it was really embarrassing. I felt ashamed, I was embarrassed, I don't know how to express this” […] Smiling wasn’t possible. And then, well yes. I kept telling myself: just keep your mouth closed, talk with your head bowed down to the ground (female participant, age 33).

### Type D: Avoiding the dentist due to fear (n = 4)^1^

Anxiety patients have developed a strong sense of fear due to various circumstances which prevent them from going to the dentist.

[...] it’s really life-threatening what you feel there. A person who doesn't have panic attacks can't imagine that. […] You are on the verge of hyperventilating; you become completely unaccountable for anything, actually (female participant, age 52).

The fear, that fear of the dentist. Only this fear is in your head, ugh, there's the sound of that drill again, oh, man (male participant, age 38).

In general, anxious behaviour can be related to the dentist and dental procedures. However, it can also be caused by the fear of disrespect and lack of respect for the anxiety problem itself. Anxiety may be triggered by a single dental encounter or by a multitude of disappointments and negative experiences at the dentist’s. Over time, dental visits are compared and linked to previous treatments, reinforcing the fear of recurrent negative experiences.

So, no, nothing can change it much. So this fear, this phobia, still remains. It's the same when someone’s afraid of spiders. You can't get rid of it. It's just there. As soon as there is a spider again, you are afraid again (male participant, age 38).

Immediately before a necessary visit to the dentist, mental and/ or physical reactions increase. Due to the highly pronounced anxiety, the visit to the dentist is postponed until it becomes unavoidable, whereby the patient may even try to delay the appointment by taking pain medication.

## Discussion

The present study aims to map dentist avoidance using a typology in order to identify out the commonalities and differences among avoiding patients in a transferable way. Due to its accuracy, the study may achieve high importance, since it captures dental care avoidance directly from the patient's perspective. To the best of our knowledge, this is the first study in Germany to be presented in the form of a typology. In addition, it is the first study to provide a deeper insight into the different manifestations of dental avoidance and the reasons for it. Last but not least, the study helps to improve our general understanding of the avoidance behaviour of dental patients, making it more comprehensible.

### Methodological strengths and limitations

With the qualitative research design it was possible to map the complexity of different avoidance facets within a sample and to expand the previous understanding of avoidance. Using the qualitative approach, the basic phenomena of avoidance could thus be perceived and understood from a different perspective. This fills the gaps in previous knowledge and clarifies previously existing context-specific ambiguities on questions of knowledge [39].

Given the qualitative research procedure, the qualitative approach and the quantitative approach had to be considered in a differentiated way. The benefit and added value of the respective process had to be understood in advance and harmonised with the research question [40]. In our case, it is postulated that, aside of the quantifying approach, access had to be found to the meaning and intention structures of the individual subjects. For this, the researcher attempts to see the action measures from the inner perspective of the person concerned [41]. The aim was to develop a more comprehensive understanding of a problem that has only been poorly understood so far and had, for the most part, only been conducted via standardised quantitative interviews. Usually, questionnaires are used with relatively large samples that leave hardly any room for self-interpretation [39]. Based on our results, collected in a patient-based manner, the study, as one of few qualitative studies in dental care avoidance behaviour to date, can make a significant contribution concerning the evidence of the understanding of avoidance [42–44]. Mainly due to the fact that the sensitive health avoidance content leads to insufficient participation in health surveys, particularly in the light of a poor health status [45], the chosen design was provided precisely for this patient group.

The use of qualitative methodological research enables a more direct and more profound reference to the social background of the study participants, which would not be the case or lead to a satisfactory evaluation using an isolated application of quantitative methodology [46, 47]. With the aid of the qualitative approach, structures that lie outside the scope of quantitative studies can be explored in depth [46]. The study design does not aim to determine a statistical value but rather to understand the reasons for avoidance and the avoidance behaviour of a small number of test participants in-depth and to present them in the form of a representative sample selection [48]. For pragmatic research reasons, it was essential that only locally available study participants were to be recruited. Accordingly, the formation of a reliable and meaningful sample was necessary. For this purpose, the principle of theoretical saturation known from grounded theory was applied, which ensures the saturation of the results after the survey stop [35].

Due to the sample design, the typology can be regarded as a valid result, which could be transferred from the avoidance ratio of North Germans to the whole of Germany. However, this would require additional studies. Furthermore, historical events, such as the division of the coutry into the Federal Republic of Germany and the German Democratic Republic WW II, seems to have produced apparent discrepancies within the two populations, which might have led to different views and characteristics concerning dentist avoidance. However, studies show that health inequalities between the two parts of Germany are almost non-existent [49]. Nevertheless, the older patients of the study may hold views that still stem from experiences with dentistry in the East German system which, at that time, was organized very differently than in West Germany [50]. The transferability of our results to other countries must also be viewed critically. Especially against the background that Germany is a so-called welfare state. In the 1990s, the Danish sociologist Gøsta Esping-Andersen's defined three types of welfare states [51]. He distinguished liberal, conservative, and social democratic welfare states. These differ in terms of the provision of social services, their quality, the impact of social measures and their social effect distribution. For example, Germany, as a conservative welfare state, offers significantly more financial support for medical treatment and preventive examinations than many other countries [52].

### New insights and possible declarations

Due to patients' different experiences and resulting perceptions, a complexity of behavioural patterns and perceptions arises in reference to dental care and access to treatment. Ultimately, however, it all leads to the same behaviour, namely avoidance. It has already been documented that fear and indifference towards dental care inevitably lead to disengagement [27]. Our results support these assumptions and show that other patient attitudes follow the same principle.

One reason often associated with dental care avoidance is anxiety. A distinction is made between pathological dental phobia, which has already been classified, and general fear of dentistry [53]. Dental phobia and dental anxiety are omnipresent in clinical practice. Accordingly, we were also able to identify patients with dental anxiety in the study, with the prior exclusion of patients with dental phobia, and to establish that fear is expressed in different ways and intensities, as has already been demonstrated by other studies [54].

Anxiety due to visits to the dentist and ensueing dental treatments has already been addressed extensively in the literature [55–57]. In addition to this predisposing view, which may be considered as too generalised concerning avoidance patients, we urgently refer to the other avoidance types that exist independent of the fear problem. Although anxiety has already been associated with avoidance due to embarrassment [26], the patients’ disengagement due to existing feelings of guilt must be considered separately. Similar to the anxiety patient, there is a mental confrontation with the problem. However, the difference in these patients is that taboo- thinking triggered by avoidance leads to the attempt of hiding one’s mouth [26]. Hereby, the disengagement is based on the patient's sense of shame and the embarrassing outward effect on others, not on the fear of the dentist or the visit to the dentist.

According to our results, disappointed patients who feel ignored by the dentist can also be considered as a separate group. From these patients' perspective, the dentist sees himself as the main actor in the treatment and does not deal enough with the patients’ expectations and ideas. This causes feelings of distrust, a lack of clarity, and the assumption that the dentist's fields of interest lie in other areas than those of the patient. A satisfactory outcome seems out of reach and may not be achieved [42]. The main problem here may be based on lack of empathy, understanding and communication [58, 59] within the treatment realm.

The group of distanced patients can also be separated. This type reflects the contents of the attitude already described in dental indifference [27]. In addition, we were able to divide the distanced type into two subtypes. Negligent patients have a comparatively strong lack of understanding of dental precautions and self-motivation. Evidence shows that this attitude is observed mainly in younger age groups and that apathy, which had been intense initially, decreased progressively in later years [60]. We support these statements,since the negligence observed in our study was likewise associated with the youngest participants (male participants, 20 and 32 years of age and female participants, 28 years of age). Also, the patients regretted their attitude, retrospectively. This shows that the careless attitude decreases with age and expands towards preventive thought. Referring to the other distanced subtype, it can be stated that the influences, which have already proved to hurt a regular oral health routine and preventive care [28, 33], correlate with the patients’ statements and are used as excuses to justify negligence and to hide one's failure.

Dental negligence has long been considered a significant behaviour of dental patients. For example, in a survey by the Office of Population Censuses and Surveys, dental apathy was cited by more than a quarter of the respondents as due to dental negligence [60]. This can be underlined by a Scottish study which, in the light of a health campaign survey, found that fear of dental treatment was a minor avoidance problem [61] compared to apathy as a barrier to seeking dental treatment. This confirms that behaviours that accompany us and are taken for granted in everyday life should not be neglected when it comes to the behaviour of avoidance. Therefore, qualitative results, such as ours, are indispensable to the development of general understanding in this field, employing currently available means for prevention. Furthermore, the use of such a basis is essential for creating suitable intervention strategies in a targeted manner.

It may be advantageous to adopt an approach of combining quantitative and qualitative research methods in the form of "mixed methods" [42]. Existing questionnaires, such as the established Dental Anxiety Scale [62] or the Dental Indifference Questionnaire [27], were designed for specific avoidance groups. These scales provide a quick method for assessing a seemingly significant and fixed group of patients and target groups in need of oral health promotion. Qualitative results will essentially enhance questionnaires of this kind. For example, the Dental Indifference Questionnaire shows that specific questions elicit the same sort of response from dentally anxious patients and dentally indifferent patients [27]. Accordingly, questionnaires, which have proven to be reasonably practicable for quantitative data collection in medicine, may become more valuable through qualitative research when the specific behavioural patterns of patients practicing avoidance can be distinguished from each other more easily, beforehand. In particular, type-casting is beneficial for this process as it can delineate characteristics from each other.

It needs to be pointed out that the already existing Dental Indifference Scale ought to be used as a supplement to the measurement of dental anxiety. This should make it possible to relate dental anxiety values to those of indifference to predict dental behaviour better and to be able to connect existing problems of avoidance patients [27]. A questionnaire option including the present avoidance typology and the questionnaires available so far would be a great advantage when recording, delimiting, classifying and possibly relating the broad range of avoidance patients. It would be possible to produce an efficient and rapid methodology applicable and practicable in practice. Such an approach is very likely to emerge as an efficient means for assessing oral health.

### Implications for research in dental care

We can state that qualitative research in the dental field allows for a significant increase in understanding [42, 44]. In general, the patient's voice has been gaining more and more acceptance and importance in the medical field and is already being placed above the doctor’s view in certain cases [63]. Therefore, patient-based study results can significantly support research when it comes to tapping into behaviour from the patient's perspective and generating adequate solution approaches based thereupon.

Our results support the assumption that even simple behavioural adjustments in the doctor-patient relationship and communication can reduce dental care avoidance [58, 59]. It has been confirmed that solid communication skills are necessary to educate, assess, and support patients appropriately [64]. Regarding our study, in particular, this shows that especially misunderstood and disillusioned patients might benefit greatly from enlightening conversations. Concepts such as the reference to communication training for physicians [65] show that this problem exists across all disciplines.

The problem of health care avoidance is well known in medicine and dentistry. Therefore, the possibility of transferring identified avoidance attitudes to the respective specialty needs to be investigated. Even in this framework, understanding is based on only very few qualitative studies. Generally, it is the quantitative studies [17, 30] which neither reflect the knowledge of avoidance by patients nor reappraise this from a direct patient perspective. From various researchers' standpoints, a multidisciplinary approach would be desirable owing to the interdisciplinary nature of the problem [57, 66, 67].

Therefore, interdisciplinary study results might assist when comparing avoidance patients across disciplines to work out possible parallels and commonalities and also differences between the avoidance structures. These could then be employed as a basis for the development of tailormade prevention and intervention strategies.

## Conclusion

Despite the general improvement in the oral health situation today, a large proportion of German citizens continues to avoid regular dental care [16, 56]. Although studies on health behaviour already exist and various study models and behavioural approaches have been established, there is a lack of understanding of how existing influences affect patient behaviour, ultimately leading to avoidance.

The present study was able to show that dental care avoidance is very complex and needs to be considered multidimensionally, taking current conditions into consideration. Already existing assumptions concerning avoidance could be confirmed but also extended. The study results made it possible to divide avoidance patients into four independent avoidance types in the form of a typology. The typology serves to enrich the understanding of avoidance and ensures an essential contribution and the possibility for prevention approaches that may later be built upon and specifically adapted. The qualitative research design process may provide significant progress in this regard due to its direct proximity to the patient [42–44]. Moreover, with its proximity to the patient, this approach offers a verifiable advantage when contacting patients who are difficult to reach [45].

## Data Availability

The data sets used and analysed in the current study are available from the corresponding author (LS) upon reasonable request.

## Footnotes

1 Due to the translation of the supporting quotations into English, there may be inconsistencies in places.
